# Longitudinal Associations between Posttraumatic Stress Disorder Severity and Personality Disorder Features among Female Rape Survivors

**DOI:** 10.3389/fpsyt.2017.00006

**Published:** 2017-02-02

**Authors:** Michelle J. Bovin, Erika J. Wolf, Patricia A. Resick

**Affiliations:** ^1^VA National Center for PTSD, Boston, MA, USA; ^2^Boston University School of Medicine, Boston, MA, USA; ^3^Duke University Medical Center, Durham, NC, USA

**Keywords:** PTSD, personality disorders, trauma, longitudinal, rape

## Abstract

This study evaluated how change in posttraumatic stress disorder (PTSD) symptoms was associated with residualized change in comorbid personality disorder (PD) features and *vice versa* over the course of 5–10 years. The sample was comprised of 79 female rape survivors who met criteria for PTSD and who were a part of a larger study examining the effects of trauma-focused therapy. PTSD was assessed with the fourth edition of the *Diagnostic and Statistical Manual of Mental Disorders* (DSM-IV) version of the Clinician-Administered PTSD Scale [CAPS-IV (1)] and PD features were assessed with the DSM-IV dimensional PD scales on the Schedule for Non-adaptive and Adaptive Personality [SNAP (2)]. PTSD symptom severity and PD features were assessed at baseline and between 5 and 10 years after completing treatment. Multiple regression analyses revealed that PTSD symptom change was related to residualized change in PD severity for paranoid, schizotypal, antisocial, borderline, avoidant, and dependent PD (βs ranged from −0.23 to −0.33; all *p*s < 0.05). In addition, for borderline and antisocial PDs, longitudinal stability of the PD was attenuated among those with greater PTSD symptom improvement (i.e., the relationship between these PDs over time was altered as a function of PTSD symptom change; βs ranged from −0.27 to −0.29; all *p*s < 0.05). Similarly, change in severity of paranoid, schizotypal, antisocial, avoidant, and obsessive–compulsive (OC) PD was associated with residualized change in PTSD symptoms (βs ranged from −0.32 to −0.41; all *p*s < 0.05), and the longitudinal stability of PTSD was attenuated as a product of change in OC PD (β = −0.27; *p* < 0.02). These findings suggest that these two sets of disorders may impact one another substantially, altering the course of even chronic, characterological conditions. This carries important clinical implications for the treatment of both PTSD and PDs.

## Introduction

Posttraumatic stress disorder (PTSD) is highly comorbid with a range of other mental health disorders. In fact, Brown et al. (3) found that of the anxiety and mood disorders, PTSD had the highest prevalence and most diverse pattern of comorbid psychopathology. Similarly, in the National Comorbidity Study, Kessler et al. (4) found that PTSD was associated with increased odds of being diagnosed with a mood disorder, an anxiety disorder, alcohol/drug abuse or dependence, and conduct disorder (4). More recent research has identified a link between psychosis and PTSD, with estimates suggesting that the prevalence of PTSD in people with psychotic disorders ranges from 12 to 29% (5, 6).

Posttraumatic stress disorder is also highly comorbid with many personality disorders (PDs). In a national epidemiological survey, Pietrzak et al. (7) found that 50% of individuals with PTSD also met criteria for at least one PD. Further, in a sample of male combat Veterans with PTSD, Dunn and her colleagues (8) found that more than 45% met criteria for at least one PD, and more than 16% met criteria for two or more PDs.

Traditionally, PDs have been conceptualized as stable and unresponsive to treatment. According to the fourth edition of the *Diagnostic and Statistical Manual of Mental Disorders* [DSM-IV (9); the version of the manual most relevant to this manuscript, although this definition has been preserved in DSM-5], a PD is “an enduring pattern of inner experience and behavior that … is *pervasive and inflexible* … is *stable over time*, and leads to distress or impairment,” [p. 629; italics added (9)]. Recent studies have offered greater optimism about the course of PDs, suggesting that PD features can improve both over time and in response to treatment (10, 11). Consistent with this, in a study designed to examine the association between PTSD treatment and change in PDs, Markowitz and his colleagues (12) found that after a short course of trauma-focused therapy, 43% of participants lost their PD diagnosis by the end of treatment. There was no association between PTSD change and PD change explicitly, which they hypothesized could be due to a limited sample size (only 35 participants had a PD at baseline), the range of PDs excluded (i.e., participants who met criteria for borderline, antisocial, schizoid, and schizotypal PD at baseline), and/or the broad level of PD improvement. However, they did find that 56% of patients with PTSD and a comorbid PD who responded to the intervention [i.e., ≥30% Clinician-Administered PTSD Scale (CAPS) improvement] lost their PD diagnosis by the 26-week follow-up. Because of the small sample size and range of PDs excluded, it is unclear whether Markowitz et al.’s results generalize to the full range of PDs or if differential patterns of co-occurring change might emerge across specific PDs.

In addition to the limited body of research focused on how PTSD change affects PDs over time, a similarly small number of studies have examined the comparable question regarding how PDs might affect PTSD change over time. The studies that do exist have focused on the predictive value of PDs at baseline on PTSD change; in general, they have suggested that PDs do not interfere with changes in PTSD [e.g., Ref. (13, 14)]. However, these studies have not tended to evaluate how PD *change* (as opposed to baseline PD features) may relate to PTSD change over time.

Despite the lack of empirical attention, there is reason to suspect co-occurring change in PTSD and PDs. Research has suggested that PTSD and PDs may share an underlying vulnerability that may contribute to both disorders. Specifically, trait negative emotionality (NEM; reflecting a tendency toward negative affect and generalized distress that is rooted in temperament) has been identified as the primary risk factor for the development of PTSD and its comorbidities [e.g., Ref. (15–17)], and may underlie a range of posttraumatic psychopathology (18), including PDs (19). Therefore, it is possible that co-occurring symptom reductions in PTSD and PD could arise from decreases in this shared underlying vulnerability. It is also possible that in response to trauma, repeated victimization, and avoidant behavior, people develop overgeneralized patterns of behavior and thinking that is diagnosed as a PD (20), but may more accurately reflect trauma-related psychopathology.

The purpose of this study was to examine how changes in PTSD and PD severity covary over time. To investigate this question, we conducted secondary data analyses on existing data from a treatment trial conducted by Resick et al. (21), comparing cognitive processing therapy (CPT) and prolonged exposure (PE) for the treatment of chronic PTSD in female rape survivors. We examined the baseline data from the trial as well as the long-term follow-up (LTFU) data collected 5–10 years after treatment (22). Previously published research from this trial found that PTSD symptoms decreased significantly as the result of trauma-focused therapy (21), and therefore, this dataset provided an excellent opportunity to examine the covariation of change in PTSD severity and PDs. We hypothesized that PD features would decrease in severity (as operationalized by a residualized change score variable, see below) among those with the greatest PTSD change (e.g., co-occurring symptom amelioration), and that the longitudinal stability of PD features would be attenuated in individuals who experienced the greatest amount of PTSD symptom improvement. That is, PD features, which are traditionally thought to be stable and intractable (9), may become less stable given PTSD symptom improvement. With respect to the related question of how PD changes are associated with PTSD changes, we similarly hypothesized that reductions in PD severity would predict decreases in PTSD severity (again operationalized as a residualized change score) and that the longitudinal stability of PTSD severity (23, 24) would be attenuated in individuals who experienced the greatest amount of PD symptom improvement. We did not make specific hypotheses about differential patterns of change as a function of each specific PD, but generally thought that PDs with greater saturation with underlying NEM would evidence the greatest co-occurring change.

## Participants and Methods

### Participants

Participants had experienced at least one completed rape and met full DSM-IV PTSD criteria. Treatment trial exclusion criteria included current substance dependence, illiteracy, suicidal/homicidal intent or parasuicidal behavior, psychosis, involvement in an abusive relationship, and being stalked. There were 171 participants in the original intent-to-treat sample. Of these, 79 participants had complete data on all variables of interest for this study. Participants who were excluded from the current study did not differ from those included on age (*t* = 0.33; *p* > 0.05), education (*t* = 0.32; *p* > 0.05), race (χ^2^ = 3.05; *p* > 0.05), or baseline PTSD severity or PD scores (all *t*s < 1.86; all *p*s > 0.05). The 79 participants ranged in age from 18 to 55 years (M = 31.7; SD = 9.6); 70% self-reported as Caucasian, 27% African-American, and 3% other. Participants reported 10–24 years of education (M = 14.3; SD = 2.3). Of the 79 included participants, 38 were randomly assigned to receive CPT and 41 to PE. This was a highly traumatized sample. In addition to experiencing at least one completed rape, the prevalence of additional trauma exposure in the sample was as follows: 39% (*n* = 31) reported childhood sexual abuse; 24% (*n* = 29) witnessed or learned of a criminal or vehicular homicide of a close friend or family member; 15% (*n* = 12) reported being the victim of attempted murder; 18% (*n* = 14) reported being robbed; 21% (*n* = 17) reported being kidnapped; 19% (*n* = 15) reported serious physical assault; and 53% (*n* = 42) reported experiencing at least one other rape (in addition to the index event).

### Procedure

Data for the current study were collected as part of a larger randomized controlled trial (21). All participants received either CPT or PE. Approximately 5–10 years later (M = 6.03 years, SD = 0.93 years), participants were re-contacted and asked to complete the same measures as at baseline. Institutional Review Board approval was secured for all phases of the study.

### Measures

The Clinician-Administered PTSD Scale for DSM-IV [CAPS-IV (1)] is a reliable and valid structured diagnostic interview that assesses DSM-IV PTSD severity and diagnosis. For each symptom, a clinician rates two dimensions, frequency and intensity, on separate five-point scales; these scales can be combined across items to form a total PTSD severity score, which was used in this study. Interrater reliability for total PTSD symptom severity was 0.97 for baseline and 0.94 for LTFU.

The Schedule for Non-adaptive and Adaptive Personality [SNAP (2)] is a personality inventory comprised of validity scales, temperament and trait scales, and 13 PD scales with dichotomous (diagnostic) and dimensional scoring options. Ten scales reflect DSM-IV PD criteria. The dimensional scores for these 10 PD scales were used in analyses because prior work suggests that they are internally consistent and relate well to interview-based PD assessments; the dichotomous scores show weaker associations with these assessments (2, 25). Further, the long-term rank-order stability (i.e., correlation) of the dimensional scores is strong [mean 2-year PD severity correlation = 0.69 (26)], even with declining group mean levels over time, suggesting that these scales tend to be stable and reliable over time. We also chose to focus on the dimensional scores rather than the diagnostic scores because this approach is more aligned with research supporting the dimensionality of PDs (25, 27, 28).

At the LTFU visit, we assessed additional treatment completed after the trial with a single yes/no question: “Have you received more therapy since you completed our program?”

### Data Analysis

First, we examined group mean changes for PDs and PTSD by comparing baseline and LTFU SNAP and CAPS scores using paired-sample *t*-tests. In addition, we conducted correlational analyses to examine how PD feature scores at each time point were associated with each other as well as with the CAPS severity scores at both time points.

To test our first hypothesis, that individuals who experienced improvement in PTSD symptoms also demonstrated reductions in PD features, we ran 10 hierarchical multiple regression analyses (1 for each PD). Each regression had five steps with the LTFU PD score as the dependent variable and the following variables entered into each step of the equation as predictors: (1) SNAP baseline PD score; (2) treatment completer status (0 = *did not complete treatment*; 1 = *completed treatment*); (3) CAPS change score (baseline-LTFU); (4) interaction of baseline PD × CAPS change; and (5) additional treatment received after study completion (0 = *no additional treatment received*; 1 = *additional treatment received*). In these analyses, the dependent variable is an index of residualized change in each PD. Residualized change scores (29, 30) are common in the research literature [e.g., Ref. (31–34)]. They reflect residual variance in the dependent variable that is not predicted by the same variable measured at an earlier time point. In other words, when there is either positive or negative residual variance in the dependent variable, this reflects increased or decreased level of the dependent variable, respectively.

Step 1 allowed for evaluation of the longitudinal stability of the PD features and controlled for baseline PD scores in subsequent steps, with a positive sign indicating that higher PD feature scores at baseline were associated with higher PD feature scores at LTFU. Step 2 controlled for the effect of treatment completion to separate variance associated with therapy completion from that associated with symptom change. Treatment completion was entered at Step 2 because we wanted to first account for variance attributable to completion of the study and then determine the extent to which PTSD symptom change accounted for additional variance beyond that attributable to simply completing the study. For this step, a negative sign indicated that completer status was associated with decreases in PD at LTFU. Step 3 tested the main effect of PTSD change on residualized change in LTFU PD features. In other words, this step examined if greater PTSD symptom improvement was associated with less PD severity at LTFU when baseline levels of the PD were held constant at the sample mean. For this step, a negative sign indicated that more PTSD symptom reduction was associated with lower LTFU PD feature scores. Step 4 tested if PTSD change moderated the relationship between baseline and LTFU PD scores. That is, this step examined if the longitudinal stability of PDs was attenuated among those with the greatest PTSD symptom improvement. This step effectively begins to look at subgroups within the sample by examining if there are different slopes for the longitudinal PD association as a function of degree of PTSD change. A negative sign for this step suggested that the stability of the LTFU PD was attenuated as a product of greater PTSD reduction. Step 5 tested whether additional treatment received after the treatment phase of the study added additional variance to the model. This was entered as the last step to reflect the temporal ordering of the variables since this variable captured variance from the end of the treatment trial to the LTFU. For this step, a positive sign indicated that individuals engaged in additional treatment after the trial phase of the study had higher LTFU PD feature scores.

We followed the same analytic approach to test our second and complementary hypothesis, that individuals who demonstrate a reduction in PD features also demonstrate decreases in PTSD symptoms (as indexed by residualized change scores), in 10 hierarchical multiple regression analyses (one for each PD). As before, each regression had five steps: (1) CAPS baseline severity score; (2) treatment completer status; (3) PD change score (baseline-LTFU); (4) interaction of baseline CAPS × PD change; and (5) additional treatment received after study completion. Interpretation of these regression coefficients mirrored those described above, with the exception being that these related to LTFU PTSD scores (not PDs).

For both sets of analyses, we used Holm (35) correction for the overall *F*-test to protect against type I error given the 10 regressions (one for each PD) conducted in each set of analyses. Effect size was evaluated by calculating Cohen’s (36) *f*^2^ (0.02 = small, 0.15 = medium, 0.35 = large). Prior to conducting hypothesis testing, we centered all predictors included in interaction terms to avoid concerns related to multicollinearity and inflated standardized effect size estimates.

## Results

Descriptive statistics for the CAPS and PD scales, and *t*-tests examining group mean changes over time, are presented in Table 1. Correlational analyses are presented in Table 2. Before proceeding with our planned analyses, we explored the potential effect of treatment type. We compared means for the two treatments at baseline and LTFU; participants who received CPT versus PE did not differ on baseline (all *t*s < 1.90; all *p*s > 0.15) or LTFU (all *t*s < 1.70; all *p*s > 0.05) CAPS or PD scores in these 79 subjects. Therefore, we collapsed the data across treatment type for all subsequent analyses.

**Table 1:** **Baseline and LTFU posttraumatic stress disorder symptom severity scores and personality disorder feature scores**.

Variable	Time point				*t*-Test *t* (df)
	Baseline		LTFU		

	(*n* = 79)				
	M	SD	M	SD	
CAPS	77.15	19.61	26.33	24.31	17.40 (78)**
SNAP					
Paranoid	13.77	4.89	8.79	5.59	9.18 (78)**
Schizoid	7.10	3.17	5.70	3.40	4.26 (78)**
Schizotypal	12.19	4.29	8.32	4.94	7.46 (78)**
Antisocial	8.97	5.44	7.57	5.25	3.04 (78)**
Borderline	11.81	4.85	8.24	5.83	6.06 (78)**
Histrionic	8.94	4.19	7.67	3.69	3.43 (78)**
Narcissistic	9.30	3.92	7.27	3.67	5.18 (78)**
Avoidant	11.11	3.76	8.33	4.48	5.86 (78)**
Dependent	9.89	4.68	6.35	4.16	7.81 (78)**
OC	12.85	3.23	10.81	3.74	5.62 (78)**

*LTFU, long-term follow-up; CAPS, Clinician-Administered PTSD Scale; SNAP, Schedule for Non-adaptive and Adaptive Personality; OC, obsessive–compulsive*.

**p < 0.01.

**Table 2:** **Correlations between baseline and LTFU posttraumatic stress disorder symptom severity scores and personality disorder scores (*n* = 79)**.

Variable	CAPS-IV	Paranoid	Schizoid	Schizotypal	Antisocial	Borderline	Histrionic	Narcissistic	Avoidant	Dependent	OC
CAPS-IV		0.37**	0.22	0.42**	0.25*	0.28*	0.15	0.22	0.28*	0.30**	0.19
Paranoid	0.14		0.62**	0.87**	0.51**	0.70**	0.15	0.58**	0.72**	0.44**	0.59**
Schizoid	−0.10	0.22		0.69**	0.27*	0.37*	−0.34**	0.18	0.76**	0.05	0.35*
Schizotypal	0.13	0.68**	0.47**		0.52**	0.68**	0.08	0.55**	0.74**	0.42**	0.53**
Antisocial	0.15	0.29**	−0.04	0.42**		0.72**	0.50**	0.58**	0.25*	0.51**	0.24*
Borderline	0.36**	0.42**	0.01	0.48**	0.64**		0.35**	0.57**	0.53**	0.66**	0.49**
Histrionic	0.26*	0.16	−0.46**	0.06	0.51**	0.46**		0.55**	−0.24*	0.37**	0.15
Narcissistic	0.01	0.45**	−0.11	0.43**	0.58**	0.46**	0.60**		0.21	0.36**	0.52**
Avoidant	0.09	0.47**	0.70**	0.49**	−0.04	0.22*	−0.44**	−0.10		0.33**	0.48**
Dependent	0.35**	0.12	−0.32**	0.08	0.17	0.49**	0.39**	0.05	0.02		0.21
OC	0.10	0.53**	0.26**	0.34**	0.03	0.27*	−0.06	0.21	0.52**	−0.05	

*The lower triangle reflects correlations from baseline assessments and the upper triangle reflects correlations from the LTFU assessments. LTFU, long-term follow-up; OC, obsessive–compulsive; CAPS-IV, Clinician-Administered PTSD Scale for DSM-IV*.

*p < 0.05.

**p < 0.001.

### Predicting LTFU PDs

Next, we evaluated if change in PTSD severity was associated with residualized change in PD features in 10 separate regressions. For all regressions, the overall *F*-test met the adjusted Holm criterion for statistical significance (all *p*s < 0.005). In each equation, baseline PD significantly predicted LTFU PD with large effect sizes (all (*f*^2^ > 0.30; Table 3), indicating substantial stability. Completion of treatment added a small amount of incremental variance to the prediction of residualized change in LTFU paranoid (*f*^2^ = 0.06), schizotypal (*f*^2^ = 0.05), and obsessive–compulsive (OC) PD (*f*^2^ = 0.04; Table 3), suggesting that completing a course of trauma-focused therapy was associated with decreased PD features at LTFU. Change in CAPS scores added significant incremental variance to the prediction of LTFU paranoid (*f*^2^ = 0.09), schizotypal (*f*^2^ = 0.12), antisocial (*f*^2^ = 0.05), borderline (*f*^2^ = 0.06), avoidant (*f*^2^ = 0.09), and dependent PD (*f*^2^ = 0.09; Table 3), suggesting that PTSD symptom reductions were associated with residualized change (reductions) in these PDs (and similarly, PTSD symptom increases were associated with PD increases).

**Table 3:** **Hierarchical linear regression analyses predicting change in PD features as a function of change in posttraumatic stress disorder**.

	Predictor				
PD	Step 1Baseline PD	Step 2Tx completer	Step 3CAPS Δ	Step 4 Baseline PD × CAPS Δ	Step 5 Additional Tx
**Paranoid**					
Δ*R*^2^	0.34**	0.06**	0.09**	0.01	0.01
β	0.58**	−0.24**	−0.29**	−0.08	0.19
*B* (SE)	0.67 (0.11)	−2.83 (1.06)	−0.06 (0.02)	−0.00 (0.00)	1.29 (0.98)
(CI)	(0.46–0.88)	(−4.94 to −0.72)	(−0.10 to −0.03)	(−0.01 to 0.00)	(−0.66 to 3.23)
**Schizoid**					
Δ*R*^2^	0.36**	0.02	0.03	0.01	0.00
β	0.60**	−0.15	−0.16	−0.11	0.05
*B* (SE)	0.65 (0.10)	−1.06 (0.66)	−0.02 (0.01)	−0.00 (0.00)	0.36 (0.63)
(CI)	(0.45–0.84)	(−2.36 to 0.25)	(−0.04 to 0.00)	(−0.01 to 0.00)	(−0.90 to 1.62)
**Schizotypal**					
Δ*R*^2^	0.26**	0.04*	0.11**	0.02	0.04*
β	0.51**	−0.22*	−0.33**	−0.14	0.20*
*B* (SE)	0.59 (0.11)	−2.25 (1.04)	−0.06 (0.02)	−0.01 (0.00)	2.04 (0.90)
(CI)	(0.36–0.81)	(−4.31 to −0.19)	(−0.10 to −0.03)	(−0.02 to 0.00)	(0.25–3.83)
**Antisocial**					
Δ*R*^2^	0.50**	0.01	0.05**	0.08**	0.01
β	0.71**	−0.12	−0.23**	−0.29**	0.08
*B* (SE)	0.68 (0.08)	−1.29 (0.91)	−0.05 (0.02)	−0.01 (0.00)	0.89 (0.82)
(CI)	(0.52–0.84)	(−3.11 to 0.53)	(−0.08 to −0.02)	(−0.02 to −0.01)	(−0.75 to 2.52)
**Borderline**					
Δ*R*^2^	0.28**	0.02	0.06**	0.07**	0.03*
β	0.53**	−0.13	−0.25**	−0.27**	0.19*
*B* (SE)	0.64 (0.12)	−1.59 (1.19)	−0.06 (0.02)	−0.01 (0.01)	2.27 (1.07)
(CI)	(0.41–0.87)	(−3.96 to 0.79)	(−0.10 to −0.01)	(−0.02 to −0.01)	(0.14–4.41)
**Histrionic**					
Δ*R*^2^	0.44**	0.01	0.00	0.00	0.00
β	0.66**	−0.10	−0.03	0.04	−0.03
*B* (SE)	0.58 (0.08)	−0.75 (0.67)	−0.00 (0.01)	0.00 (0.00)	−0.26 (0.70)
(CI)	(0.43–0.73)	(−2.08 to 0.58)	(−0.03 to 0.02)	(−0.01 to 0.01)	(−1.65 to 1.12)
**Narcissistic**					
Δ*R*^2^	0.33**	0.00	0.03	0.01	0.00
β	0.58**	−0.05	−0.18	0.07	0.07
*B* (SE)	0.54 (0.09)	−0.40 (0.73)	−0.03 (0.01)	0.00 (0.00)	0.51 (0.75)
(CI)	(0.37–0.71)	(−1.85 to 1.04)	(−0.05 to 0.00)	(−0.00 to 0.01)	(−0.99 to 2.01)
**Avoidant**					
Δ*R*^2^	0.24**	0.01	0.08**	0.02	0.01
β	0.49**	−0.11	−0.29**	−0.15	0.12
*B* (SE)	0.58 (0.12)	−1.06 (0.95)	−0.05 (0.02)	−0.01 (0.01)	1.14 (0.88)
(CI)	(0.34–0.82)	(−2.95 to 0.84)	(−0.08 to −0.02)	(−0.02 to 0.00)	(−0.63 to 2.90)
**Dependent**					
Δ*R*^2^	0.35**	0.03	0.09**	0.00	0.01
β	0.59**	−0.16	−0.30**	−0.05	0.08
*B* (SE)	0.53 (0.08)	−1.39 (0.80)	−0.05 (0.01)	−0.00 (0.00)	0.65 (0.74)
(CI)	(0.36–0.69)	(−2.98 to 0.20)	(−0.08 to −0.02)	(−0.01 to 0.00)	(−0.83 to 2.13)
**OC**					
Δ*R*^2^	0.34**	0.04*	0.02	0.00	0.01
β	0.58**	−0.19*	−0.14	−0.01	0.08
*B* (SE)	0.67 (0.11)	−1.53 (0.73)	−0.02 (0.01)	0.00 (0.00)	0.63 (0.72)
(CI)	(0.46–0.88)	(−2.98 to −0.07)	(−0.05 to 0.01)	(−0.01 to 0.01)	(−0.80 to 2.06)

*PD, personality disorder; CAPS, Clinician-Administered PTSD Scale; Tx, treatment; OC, obsessive–compulsive; Δ*R*^2^, *R*-squared change; β, standardized beta; *B*, unstandardized beta; CI, 95% confidence interval for B. All overall *F*-tests were significant using Holm’s adjusted *p*-value thresholds (all *F*s > 8.47; all *p*s < 0.001)*.

*For illustrative purposes, only the final variable added to each step is displayed in the table. The dependent variable for each regression was the respective long-term follow-up PD feature score. PDs are listed in the first column, as opposed to the top row (which might be more typical) due to space considerations*.

*p < 0.05.

**p < 0.01.

**Figure 1:**
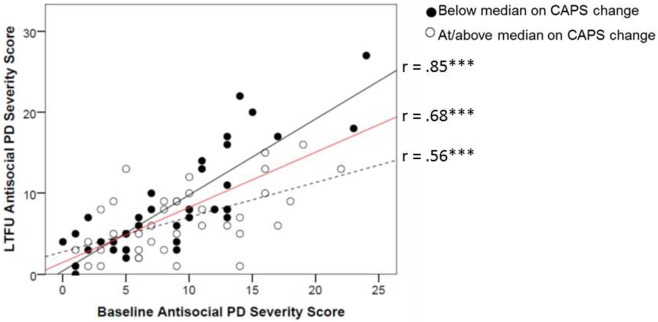
**Change in mean raw antisocial PD score from baseline to LTFU as a function of improvement in CAPS score from baseline to LTFU**. Degree of improvement on the CAPS was defined by a median split on the baseline minus LTFU difference score (median CAPS change score = 50). The red line reflects the average stability for this PD for the sample. CAPS, Clinician-Administered PTSD Scale; PD, personality disorder; LTFU, long-term follow-up. ^+^*p* < 0.10; *p < 0.05; **p < 0.01; ***p < 0.001.

**Figure 2:**
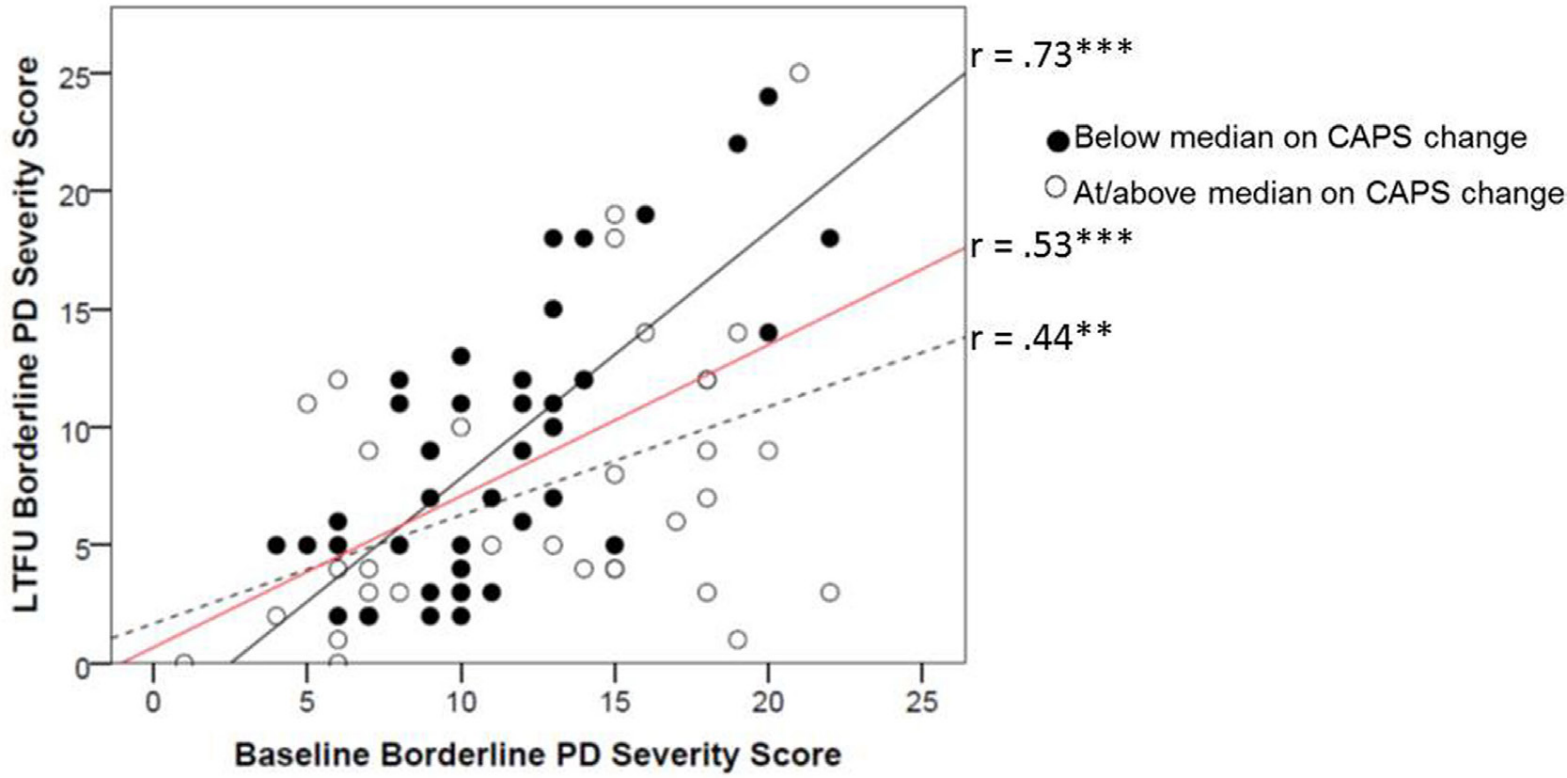
**Change in mean raw borderline PD score from baseline to LTFU as a function of improvement in CAPS score from baseline to LTFU**. Degree of improvement on the CAPS was defined by a median split on the baseline minus LTFU difference score (median CAPS change score = 50). The red line reflects the average stability for this PD for the sample. CAPS, Clinician-Administered PTSD Scale; PD, personality disorder; LTFU, long-term follow-up. ^+^*p* < 0.10; *p < 0.05; **p < 0.01; ***p < 0.001.

The interaction between baseline PD features and CAPS change added incremental variance in Step 4 to the prediction of LTFU antisocial (*f*^2^ = 0.09) and borderline PD (*f*^2^ = 0.07; Table 3). To depict these interactions, we plotted the association between PD at baseline and LTFU as a function of change in CAPS. Degree of CAPS change was defined by a median split on the CAPS difference score for the purposes of the figures (Figures 1 and 2). These figures show that participants with greater decreases on the CAPS evidenced sharper declines in PD features over time (i.e., the substantial stability of PD features evident from the results of Step 1 of the equation was attenuated for individuals who achieved greater PTSD improvement). These figures also show the stability (i.e., slope) of borderline and antisocial PD change as a function of CAPS change in comparison to the average stability of these PDs over time (shown in red). Results indicated that additional treatment after completion of the study treatment protocol resulted in small but significant increases in variance explained in LTFU schizotypal (*f*^2^ = 0.04) and borderline PD (*f*^2^ = 0.03; Table 3), such that participants who sought additional treatment had higher LTFU PD scores than those who did not.

In summary, this set of analyses revealed that greater PTSD symptom improvement was associated with greater residualized change (symptom improvement) in paranoid, schizotypal, antisocial, borderline, avoidant, and dependent PDs. Further, results revealed that for borderline and antisocial PD, the stability of the PD was not uniform across subjects, but rather, there were different slopes for the longitudinal PD association that differed by the degree of PTSD change.

### Predicting LTFU PTSD

We next evaluated if change in PD features was associated with residualized change (e.g., improvements) in PTSD severity in 10 separate regressions.^1^ Five of the overall *F*-tests did not meet the Holm criterion for statistical significance and were therefore not interpreted.^2^ In each of the five equations that were interpretable (i.e., those involving paranoid, schizotypal, antisocial, avoidant, and OC PD), baseline CAPS significantly predicted LTFU CAPS (*f*^2^ = 0.06; Table 4). By contrast, completion of treatment, entered in Step 2, did not add significant incremental value to any of the models (all *p*s > 0.05). However, changes in all five of these PDs (all *f*^2^ > 0.10) added significant incremental variance to the prediction of LTFU CAPS. These results suggest that in these five analyses, PD change covaried with residualized PTSD change.

**Table 4:** **Hierarchical linear regression analyses predicting change in posttraumatic stress disorder severity as a function of change in PD features**.

	Predictor				
PD	Step 1Baseline CAPS	Step 2Tx completer	Step 3PD Δ	Step 4Baseline CAPS × PD Δ	Step 5Additional Tx
**Paranoid**					
Δ*R*^2^	0.053*	0.036	0.16***	0.027	0.0020
β	0.23*	−0.19	−0.41***	−0.17	−0.041
*B* (SE)	0.26 (0.13)	−8.23 (4.93)	−1.71 (0.45)	−0.038 (0.024)	−1.69 (4.46)
(CI)	(0.001–0.51)	(−18.06 to 1.59)	(−2.60 to −0.82)	(−0.086 to 0.010)	(−10.59 to 7.22)
**Schizoid^a^**					
Δ*R*^2^	0.053*	0.036	0.062*	0.018	0.000
β	0.23*	−0.19	−0.25*	−0.15	0.001
*B* (SE)	0.26 (0.13)	−8.23 (4.93)	−1.74 (0.77)	−0.056 (0.045)	0.049 (4.74)
(CI)	(0.001–0.51)	(−18.06 to 1.59)	(−3.28 to −0.20)	(−0.15 to 0.035)	(−9.4 to 9.5)
**Schizotypal**					
Δ*R*^2^	0.053*	0.036	0.15***	0.014	0.006
β	0.23*	−0.19	−0.40***	−0.14	−0.083
*B* (SE)	0.26 (0.13)	−8.23 (4.93)	−1.74 (0.46)	−0.028 (0.024)	−3.45 (4.64)
(CI)	(0.001–0.51)	(−18.06 to 1.59)	(−2.66 to −0.82)	(−0.76 to 0.020)	(−12.71 to 5.81)
**Antisocial**					
Δ*R*^2^	0.053*	0.036	0.13***	0.005	0.003
β	0.23*	−0.19	−0.36***	−0.077	−0.056
*B* (SE)	0.26 (0.13)	−8.23 (4.93)	−1.76 (0.53)	−0.017 (0.027)	−2.32 (4.66)
(CI)	(0.001–0.51)	(−18.06 to 1.59)	(−2.81 to −0.71)	(−0.070 to 0.036)	(−11.61 to 6.97)
**Borderline^a^**					
Δ*R*^2^	0.053*	0.036	0.050*	0.016	0.002
β	0.23*	−0.19	−0.23*	−0.14	−0.043
*B* (SE)	0.26 (0.13)	−8.23 (4.93)	−0.88 (0.44)	−0.028 (0.025)	−1.77 (4.99)
(CI)	(0.001–0.51)	(−18.06 to 1.59)	(−1.75 to −0.009)	(−0.078 to 0.022)	(−11.73 to 8.18)
**Histrionic^a^**					
Δ*R*^2^	0.053*	0.036	0.001	0.012	0.000
β	0.23*	−0.19	0.027	−0.11	−0.001
*B* (SE)	0.26 (0.13)	−8.23 (4.93)	0.18 (0.79)	−0.044 (0.046)	−0.044 (4.98)
(CI)	(0.001–0.51)	(−18.06 to 1.59)	(−1.39 to 1.76)	(−0.14 to 0.048)	(−9.98 to 9.89)
**Narcissistic^a^**					
Δ*R*^2^	0.053*	0.036	0.027	0.030	0.000
β	0.23*	−0.19	−0.16	−0.17	0.011
*B* (SE)	0.26 (0.13)	−8.23 (4.93)	−1.01 (0.69)	−0.054 (0.035)	0.45 (4.81)
(CI)	(0.001–0.51)	(−18.06 to 1.59)	(−2.39 to 0.37)	(−0.13 to 0.016)	(−9.16 to 10.05)
**Avoidant**					
Δ*R*^2^	0.053*	0.036	0.12*	0.027	0.001
β	0.23*	−0.19	−0.35*	−0.18	−0.039
*B* (SE)	0.26 (0.13)	−8.23 (4.93)	−1.72 (0.54)	−0.050 (0.032)	−1.63 (4.70)
(CI)	(0.001–0.51)	(−18.06 to 1.59)	(−2.80 to −0.65)	(−0.11 to 0.014)	(−11.00 to 7.74)
**Dependent^a^**					
Δ*R*^2^	0.053*	0.036	0.013	0.057*	0.001
β	0.23*	−0.19	−0.13	−0.25*	0.036
*B* (SE)	0.26 (0.13)	−8.23 (4.93)	−0.65 (0.64)	−0.07 (0.032)	1.50 (4.88)
(CI)	(0.001–0.51)	(−18.06 to 1.59)	(−1.94 to 0.63)	(−0.13 to −0.005)	(−8.24 to 11.24)
**OC**					
Δ*R*^2^	0.053*	0.036	0.094**	0.065*	0.000
β	0.23*	−0.19	−0.32**	−0.27*	0.013
*B* (SE)	0.26 (0.13)	−8.23 (4.93)	−1.99 (0.70)	−0.085 (0.035)	0.53 (4.58)
(CI)	(0.001–0.51)	(−18.06 to 1.59)	(−3.40 to −0.59)	(−0.16 to −0.015)	(−8.61 to 9.67)

*PD, personality disorder; CAPS, Clinician-Administered PTSD Scale; Tx, treatment; OC, obsessive–compulsive; ΔR^2^, R-squared change; β, standardized beta; B, unstandardized beta; CI, 95% confidence interval for B*.

*For illustrative purposes, only the final variable added to each step is displayed in the table. The dependent variable for each regression was long-term follow-up CAPS score*.

*^a^The overall F-tests for these five analyses were not significant after applying the adjusted Holm p-value threshold; therefore, results of these analyses are not interpreted in the manuscript*.

*p < 0.05.

**p < 0.001.

***p < 0.001.

**Figure 3:**
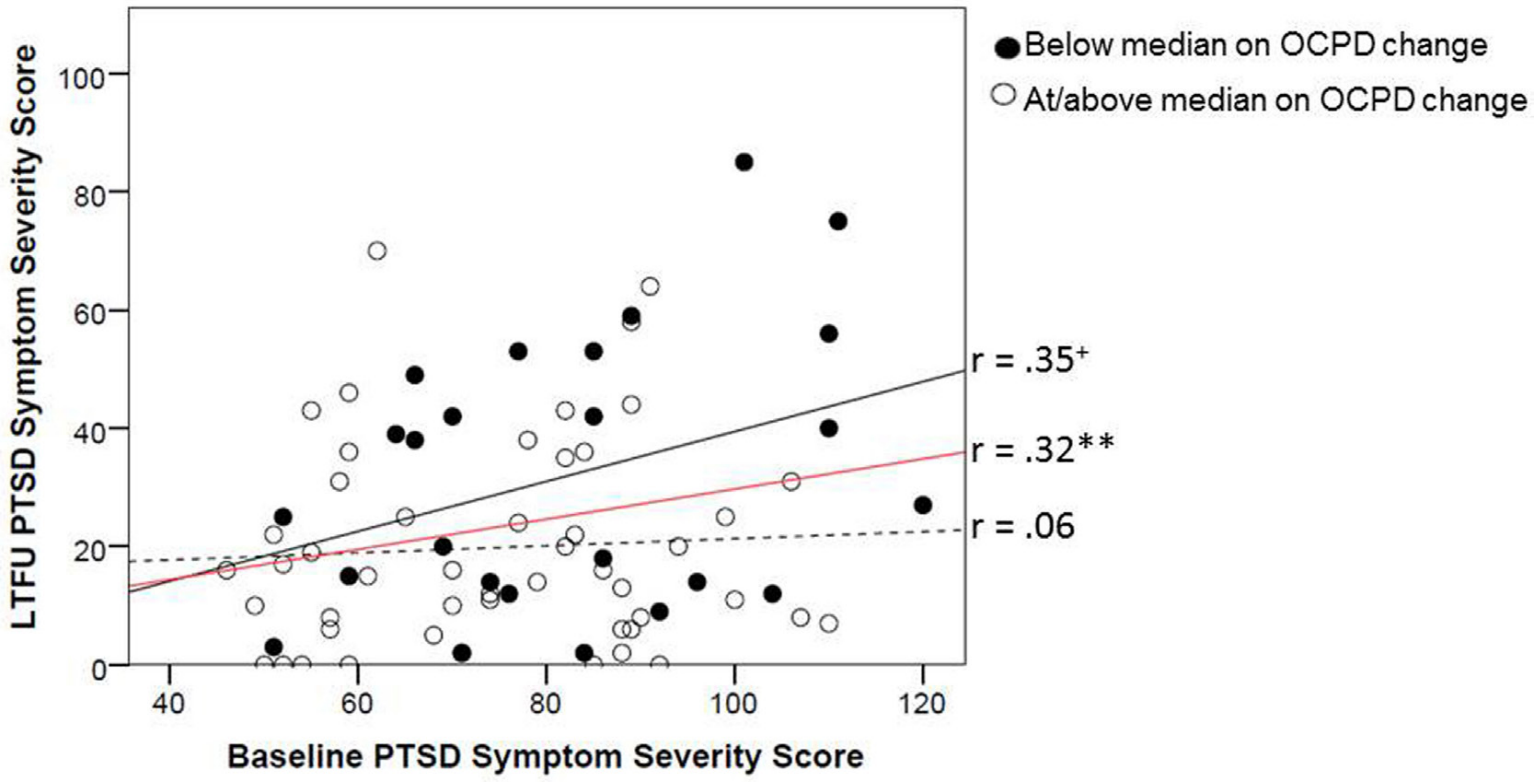
**Change in mean raw CAPS score from baseline to LTFU as a function of improvement in OC PD score from baseline to LTFU**. Degree of improvement on OC PD was defined by a median split on the baseline minus LTFU difference score (median OC PD change score = 1). OC, obsessive–compulsive; CAPS, Clinician-Administered PTSD Scale; PD, personality disorder; LTFU, long-term follow-up. ^+^*p* < 0.10; *p < 0.05; **p < 0.01; ***p < 0.001.

The interaction between baseline CAPS and change in OC PD features (*f*^2^ = 0.07) added significant incremental variance to the prediction of LTFU CAPS (Table 4). To depict this interaction, we plotted the association between baseline and LTFU PTSD symptom severity as a function of change in OC PD. Degree of PD change was defined for the purposes of the figure, by a median split on the OC PD difference score (Figure 3). The figure shows that individuals with greater decreases on OC PD evidenced greater residualized change (e.g., sharper declines) in PTSD severity over time. Specifically, the association between baseline and LTFU CAPS was attenuated for those who demonstrated the greatest OC PD improvement. The differential strength of association between PTSD at baseline and LTFU as a function of OC PD change can be viewed in contrast to the average stability of PTSD over time (shown by the red line in Figure 3). Results from Step 5 of the equations indicated that receiving additional treatment after completion of the study treatment protocol did not improve prediction of LTFU CAPS severity in any of the models (Table 4).

In summary, this set of analyses revealed that decreases in features of paranoid, schizotypal, antisocial, avoidant, and OC PD were associated with greater residualized change (decreased severity) in PTSD. Beyond this, the average stability of PTSD symptoms over time was altered as a function of the degree of change in OC PD features, with subjects who showed greater decreases in this PD also demonstrating reduced PTSD stability over time.

## Discussion

To our knowledge, the current study is the first to examine associations between PTSD and PD symptom change over the long-term and to do so for all 10 DSM-IV PDs. To explore these associations, we conducted two sets of analyses. Our first set of analyses examined how change in PTSD severity was associated with residualized change in PD severity. We found that change in PTSD severity correlated with PD scores at LTFU for six of the PDs (paranoid, schizotypal, antisocial, borderline, avoidant, and dependent), even after controlling for baseline levels of these PDs. This association implies that PTSD change was associated with residualized change in these six PDs such that improvements in PTSD covaried with reductions in each PD. This extends the work of Markowitz et al. (12) in that our results suggest that PTSD change is associated with PD change, and that participation in trauma-focused therapy is associated with decreases across a range of PDs (i.e., lower scores than what would be predicted based on baseline level of PD). These findings are particularly important because the treatments given were brief with only 13 hours of therapist contact in total conducted twice a week.

Interestingly, five of the six PDs (paranoid, schizotypal, antisocial, borderline, and dependent) that were significantly associated with change in PTSD severity in this study have been previously shown to be strongly related to trait NEM (19). The sixth PD, avoidant PD, is thought to have equivalent associations with NEM and trait positive emotionality [PEM; i.e., a tendency towards positive affect and social closeness (25)]. This suggests that the co-occurring reductions in PTSD symptom severity and these five PDs may be related to more general decreases in trait NEM which would be expected to broadly influence the expression and severity of these disorders. By contrast, three of the four PDs that were not predicted by changes in PTSD severity (schizoid, histrionic, and narcissistic PD) are most strongly associated with PEM. Though speculative, this may imply that reductions in NEM (e.g., perhaps in response to treatment) have a greater generalized impact on symptom improvements; while by contrast, PEM, which is orthogonal to NEM (37) may be unchanged as a function of treatment. Reduced NEM may be a mechanism responsible for decreases in both PTSD and the six PDs that were associated with PTSD change.

Our results further suggest that the longitudinal stability of both antisocial and borderline PD features was attenuated in individuals who experienced the greatest amount of PTSD symptom improvement. Interestingly, both of these PDs load strongly on the latent dimension of “externalizing” (18). Externalizing disorders are associated with impulse control problems (38, 39). Externalizing PDs are associated with difficulty controlling impulses toward aggressive, reckless, and dangerous behavior, and this type of behavior may perpetuate stress and lead to chronic PTSD symptoms. Given this, our finding that symptoms of these two PDs were attenuated in those with PTSD symptom reductions highlights the critical importance of treating PTSD even in the context of these particularly impairing comorbidities. Clinicians who view comorbid borderline and antisocial PD as contraindications for PTSD treatment may want to consider re-evaluating this stance.

Our second set of analyses explored how changes in PD severity might longitudinally relate to PTSD severity. For these analyses, we expected PTSD severity to evidence residualized decreases in association with decreases in PD severity, and that the longitudinal stability of PTSD severity would be attenuated in individuals who experienced the greatest amount of PD symptom improvement. Consistent with predictions, PTSD severity at LTFU was associated with changes in five PDs (paranoid, schizotypal, antisocial, avoidant, OC PD), at mean levels of baseline PTSD severity. Again, because paranoid, schizotypal, and antisocial PD are strongly associated with NEM (25), and both avoidant and OC PD are equally associated with NEM and PEM (25), these findings potentially provide additional support to the conjecture that NEM may be a mechanism responsible for change in both PTSD and these PDs. Our finding that change in OC PD was associated with attenuated longitudinal stability of PTSD symptoms is an intriguing one. One possible explanation for this may be that reductions in rigidity, a hallmark feature of OC PD, may be associated with improved cognitive flexibility, which would be expected to aide in the ability to reappraise negative trauma-related cognitions. This may be one pathway by which changes in this PD relate to residualized changes in PTSD.

The consistent association between change in PTSD and change in PDs observed in this study suggests that these two sets of disorders may be associated with one another in a more fundamental way than originally thought. It is possible that PDs may develop out of trauma exposure and PTSD symptoms. For example, assault survivors may believe that they are at fault for being assaulted and that they are incapable of making good decisions; in turn, they may become dependent upon others to make decisions for them and may develop dependent PD. Similarly, assault survivors may believe that it is not safe to trust anyone; this may develop into paranoid PD. The PD features may therefore partially represent trauma-related biases and overgeneralized schemas [sometimes referred to as overaccomodation (20, 40)], which is targeted in CPT. In this study of women with an index rape trauma, a large number had also experienced childhood abuse. Such abuse, early in personality development, could result in PTSD symptoms, as well as emotions, behaviors, and styles of relating to others that become seemingly entrenched and characterological. Therefore, trauma may be a risk factor linking PTSD and PD comorbidity, which implies that treating trauma and associated symptoms may reduce severity of both sets of disorders.

Our findings may also be relevant to individuals suffering from comorbid PTSD and psychotic symptoms. Traditionally, patients with comorbid PTSD and psychosis are not provided trauma-focused therapy (41, 42) due to the argument that treatment of PTSD in patients with psychotic symptoms is contraindicated [e.g., Ref. (41)]. Research suggests that schizoid, schizotypal, and paranoid PD may all be on the “schizophrenic spectrum” (43) and as a result, we were particularly intrigued by evidence in this study that change in these PDs was associated with change in PTSD and *vice versa*. Specifically, our results suggested that changes in PTSD were associated with both LTFU paranoid and schizotypal PD scores, even after controlling for baseline levels of these PDs. This implies that PTSD treatment in those with psychotic-spectrum disorders [i.e., paranoid, schizotypal, and schizoid PD (27, 28)] is not contraindicated, and further, that such treatment may assist in reducing symptoms of both disorders. In addition, our finding that change in these PDs also affects residualized change in PTSD severity, and that individuals who demonstrate larger decreases in paranoid and schizoid PD also demonstrate larger decreases in PTSD symptoms, suggests that targeting psychotic-like features in therapy may also reduce PTSD symptoms.

Like all studies, the current study was not without limitations. First, the study was limited in that our analyses were based on data from only two widely spaced time points (5–10 years apart). Therefore, it is unclear whether unmeasured intervening factors affected the results, although our follow-up analyses further suggest that our results cannot be explained completely by additional therapy received after the completion of trauma-focused therapy. Nevertheless, future studies should replicate these analyses with multiple time points, including an earlier follow-up, to more fully evaluate the association between PTSD and PDs. Second, with only two equally spaced data points, we could not fully examine potential reciprocal or bidirectional associations in the data or examine if change in PTSD symptoms had a greater effect on PDs relative to the effects of change in PDs on subsequent PTSD symptoms. For this reason, we conducted parallel sets of analyses for PTSD and PDs, but we are limited in our ability to draw clear causal inferences. Third, this study examined a relatively small number of women who had experienced at least one rape (although they were a severely traumatized sample). To examine if the results are generalizable to other PTSD populations, future investigations should examine whether men and patients exposed to other kinds of trauma demonstrate the same pattern of results. Finally, although we included a self-report measure of PDs that has demonstrated high construct validity with interview measures in the assessment of PDs, we did not use an interview measure designed to diagnose PDs. Future studies should use structured diagnostic interviews to assess PDs and determine if results generalize to diagnostic threshold-level PDs.

In conclusion, the current study suggests that changes in PTSD are associated with changes in a range of PDs, and conversely, changes in a number of PDs are associated with changes in PTSD symptom severity over time. Our findings raise the possibility of a shared underlying mechanism driving the covariation of change: NEM. These results have important clinical implications, in that they suggest that therapeutic efforts to reduce one set of these disorders may impact the symptomotology of the other. This runs counter to the common conceptualization that PDs are intractable and that they are contraindications for trauma-focused PTSD treatment. Thus, results provide confidence that even severe psychopathology and comorbidity may be meaningfully ameliorated, thereby reducing the considerable personal, societal, and financial burden of these disorders. Because there are short, evidence-based protocols for PTSD available, this may be the best place to start.

## Ethics Statement

This study was carried out in accordance with the recommendations of Institutional Review Board of both the University of Missouri-St. Louis and the VA Boston Healthcare System, with written informed consent from all subjects. All subjects gave written informed consent in accordance with the Declaration of Helsinki. The protocol was approved by both IRBs.

## Author Contributions

MB, EW, and PR all made substantial contributions to the conception of the work; all contributed to drafting the work, revising it critically, and provided final approval of the version to be published; all agreed to be accountable for all aspects of the work in ensuring that questions related to accuracy or integrity of any part of the work are appropriately investigated and resolved. PR acquired the data, and MB and EW were responsible for analysis and interpretation.

## Conflict of Interest Statement

The authors declare that the research was conducted in the absence of any commercial or financial relationships that could be construed as a potential conflict of interest.

## Footnotes

^1^An initial examination of the data suggested the existence of a number of potential outliers. We tested for multivariate outliers using leverage tests and found that five participants had leverage values that were more than 2 SDs above the mean. These five participants were therefore excluded from all subsequent analyses.

^2^For these analyses, the most significant test needed to surpass adjusted *p* < 0.005 (0.05/10) and the least significant test needed to surpass *p* < 0.05 (0.05/1). Overall model *p*-values for histrionic, narcissistic, dependent, borderline, and schizoid PDs did not meet these thresholds, though results are reported in Table 4 for completeness. For the remaining five analyses, the overall model surpassed the intervening adjusted *p*-value thresholds (all *p*s < 0.004).
